# Antioxidant Enzyme, Transcriptomic, and Metabolomic Changes in Lily (*Lilium* spp.) Leaves Induced by *Aphis gossypii* Glover

**DOI:** 10.3390/genes15091124

**Published:** 2024-08-26

**Authors:** Lihong Zhou, Erli Wang, Yingdong Yang, Panpan Yang, Leifeng Xu, Jun Ming

**Affiliations:** 1 State Key Laboratory of Vegetable Biobreeding, Institute of Vegetables and Flowers, Chinese Academy of Agricultural Sciences, Beijing 100081, China; vickyzlh@163.com (L.Z.); yangpanpan@caas.cn (P.Y.); xuleifeng@caas.cn (L.X.); 2 Plant Protection College, Shenyang Agricultural University, Shenyang 110866, China; wangerli1999@163.com; 3 Flower Institution, Liaoning Academy of Agricultural Sciences, Shenyang 110161, China; yangyingdong2011@163.com

**Keywords:** aphid infestation, *Aphis gossypii* Glover, induced response, lily, multi-omics

## Abstract

Cotton aphids (*Aphis gossypii* Glover) cause harm by feeding on phloem sap and spreading plant viruses to lily. Understanding the mechanisms by which aphids infest lily plants is crucial for effective aphid management and control. In this study, we investigated the activity of antioxidants, integrated nontargeted metabolomes and transcriptomes of lilies infested by cotton aphids to explore the changes in lily leaves. Overall, the results indicated that the catalase (CAT) activity in the leaves of the lily plants was greater than that in the leaves of the control plants. A comprehensive identification of 604 substances was conducted in the leaves. Furthermore, the differentially abundant metabolite analysis revealed the enrichment of phenylalanine metabolism and α-linolenic acid metabolism. Moreover, 3574 differentially expressed genes (DEGs), whose expression tended to increase, were linked to glutathione metabolism and phenylpropanoid biosynthesis. In addition, the integrated analysis revealed that the defensive response of lily leaves to aphids is manifested through antioxidant reactions, phenylpropane and flavonoid biosynthesis, and α-linolenic acid metabolism. Finally, the key metabolites were CAT, glutathione, coumaric acid, and jasmonic acid, along with the key genes chalcone synthase (*CHS*), phenylalanine ammonia-lyase (*PAL*), and 12-oxo-phytodienoic acid reductase (*OPR*). Accordingly, the findings of this research elucidate the molecular and metabolic reactions of *A. gossypii* in lily plants, offering valuable insights for developing aphid resistance strategies in lily farming.

## 1. Introduction

The cotton aphid *A. gossypii* (Hemiptera, Aphididae) is a destructive polyphagous pest that feeds on phloem and attacks more than 200 crop, garden, and wild plants, causing severe economic losses worldwide [1,2]. When aphids feed, they release proteins, metabolites, pathogenic bacteria, and viruses into the host plant [3,4,5]. This insect can inflict direct damage by continuously consuming sap from plant vascular tissues, leading to various physiological disorders and facilitating the transmission of >75 plant viruses. Moreover, the pest inhibits the photosynthetic ability of the plants through the secretion of abundant honeydew, creating an optimal environment for the growth of sooty molds [6].

The lily (*Lilium* spp.) is one of the best known ornamental plants for cut flowers, gardens, and container plantings and is the leading bulbous crop worldwide. Additionally, this plant has culinary and medicinal applications [7]. During lily production, *A. gossypii* threatens the lily industry. The lily virus disease, which is transmitted primarily by aphids, is one of the most severe diseases affecting lilies [8]. Chemical control techniques are currently the primary strategies used to avert and manage lily aphids [9]. Nonetheless, the utilization of these substances for an extended period is not favorable due to the emergence of insecticide resistance [10], suggesting that effective alternative control methods are essential and of high priority [11]. A thorough understanding of resistance mechanisms in plants serves as the cornerstone of pest management. Therefore, understanding the interactions between aphids and lily plants plays a pivotal role in infection control.

An increased research focus in recent years on plant–aphid interactions has resulted in some insights into the defense mechanisms in response to aphid attacks [12,13]. Plants can recognize phytophagous insect-related molecular patterns [14,15,16] and trigger early signaling events and hormone signaling pathways [17], resulting in transcriptome and metabolome recombination, increased direct and indirect defense compound content [18], and increased resistance to phytophagous insects. When plants respond to insect feeding, the reactive oxygen species (ROS) signal serves as the first central signal, and the physiological indicators associated with stress resistance often change. Superoxide dismutase (SOD), peroxidase (POD), and catalase (CAT) are the main antioxidant enzymes in plants, which can remove excess free radicals from the body, thereby improving the resistance of plants [19,20]. Glutathione is an essential metabolite found in eukaryotes, and it plays a crucial role in protecting cells from oxidative harm [21]. Glutathione decreased in *Zea mays* plants infected by the rose-grass aphid (*Methopolophium dirhodum* Walk.) in all studied cultivars, but glutathione decreased the most in the resistant cultivar [22]. The induced anti-insect response of plants eventually leads to increases in the expression levels of defense genes and the accumulation of defense compounds, ultimately increasing plant resistance to insects [23]. The use of combined genetic transcription and metabolic process data is a common approach for assessing plant responses to insect damage [24,25]. Plant interactions with aphids have been extensively studied at the molecular level, demonstrating that aphids provoke changes in gene expression within their host plants [26,27,28,29]. Aphid feeding can affect the expression of genes involved in cell wall formation, plant hormone pathways, oxidative stress responses, photosynthesis, and the regulation of transcription through specific transcription factor families, such as WRKY, MYB, AP2/ERF and GRAS [30]. Overall, the research cited here revealed significant alterations in plant transcriptomes and metabolomes in response to insect feeding. Although RNA sequencing (RNA-seq) and metabolic analysis have effectively revealed interactions between aphids and their hosts in various plant species [31,32], investigations into the relationships between aphids and lilies are lacking.

Naturally, resistant crop germplasms are important resources for managing agricultural product safety and environmental deterioration. In previous studies, we reported that the lily cultivar Fangio, derived by hybridizing *Lilium longiflorum* and Asiatic lily, was highly resistant to aphids, and we used Fangio plants as experimental materials [33]. In the present study, we combined antioxidant enzyme activities and gene expression analysis via high-throughput RNA-seq with metabolite profile information, coupled with bioinformatics tools, to reveal an intricate network of lily responses to *A. gossypii* herbivory. Fangio leaves were exposed to *A. gossypii* for different durations, and statistical methods were used to identify trends in the resulting transcriptomic and metabolomic datasets. Overall, examining antioxidant enzyme activities and the transcriptomic and metabolomic changes caused by *A. gossypii* infestation revealed the molecular mechanisms involved in lily defense against aphids. These results provide a better understanding of lily defense mechanisms against *A. gossypii* and a theoretical basis for the future breeding of lily varieties with increased aphid resistance.

## 2. Materials and Methods

### 2.1. Plant Growth and Insect Colony

The lily cultivar Fangio, a hybrid derived from *Lilium longiflorum* and Asiatic lily that is highly resistant to aphids, was used in this study. Lily bulbs from Haining Yihua Horticulture Limited Company (Haining, China) were stored at −1 °C. Bulbs were planted in a box (60 cm × 40 cm × 20 cm) with six bulbs in a greenhouse (26 ± 3 °C, 85 ± 10% RH, 14 L: 10 D) at the Liaoning Academy of Agricultural Sciences (41°29′ N, 123°32′ E), Shenyang, Liaoning Province, China. All healthy lily plants exhibiting similar developmental stages were watered weekly and utilized for the experiments four weeks after transplantation.

The *A. gossypii* colony originated from lily fields at the Liaoning Academy of Agricultural Sciences (41°29′ N, 123°32′ E), Shenyang, Liaoning Province, China. Colonies were reared on lily Siberian, and the population used in the present study was established under the following conditions: light, 14 L:10 D; temperature, 25 ± 1 °C; and humidity, 60% ± 10%. Second-instar larvae were used for the trials.

### 2.2. Insect Bioassays

A gentle bristle was used to carefully position twenty 2nd-instar *A. gossypii* onto the top leaf of each plant. The larvae were deprived of food for 4 h before being confined to the lily plants. The whole plants were isolated from each other with a repellent cover (60-mesh insect-proof net). After exposure to *A. gossypii*, the young lily leaves primarily damaged by the larvae were harvested for analysis of antioxidant enzyme activities (0, 24, 36, 48, 60 h) and transcriptomic and metabolomic analyses (0, 24, or 48 h), with plants at 0 h serving as controls. Then, the plant samples were immediately frozen in liquid nitrogen and stored at −80 °C for future use. For antioxidant enzyme activity measurements, six replicates were collected at the following time intervals: 0, 24, 36, 48, and 60 h. For both the transcriptomic and metabolomic analyses, three and six replicates were collected at 0, 24, and 48 h, respectively.

### 2.3. Antioxidant Enzyme Activity Measurements

The activities of plant CAT (visible light) (A007-1-1) and POD (A084-3-1), as well as total SOD (hydroxylamine method) (A001-4-1), were assessed via kits sourced from Nanjing Jiancheng Bioengineering Institute (Nanjing, China). The extraction of plant enzyme activities involved homogenizing 0.5 g of lily leaf tissue.

### 2.4. Metabolomic Analyses

Leaf samples (100 mg) from 6 biological replicates were sent for analysis of the nontargeted metabolome to LC-Bio Technology Co., Ltd., which is located in Hangzhou, China. The samples were ground with liquid nitrogen and subsequently extracted with 120 μL of precooled 50% methanol buffer. The specific extraction and mass spectrometry analysis methods used were previously described [34].

XCMS software (version 3.2) was used to preprocess the LC-MS data. We used the Kyoto Encyclopedia of Genes and Genomes (KEGG) and the Human Metabolome Database (HMDB) online databases for metabolite annotation. If the mass difference between the observed value and the database value was less than 10 ppm, the metabolite was annotated, and the molecular formula of the metabolite was further identified and validated via isotopic distribution measurements. We also used an in-house fragment spectrum library of metabolites to validate the identified metabolites. To normalize all the samples, the probabilistic quotient normalization (PQN) algorithm was used. Additionally, quality control (QC) samples were subjected to QC-robust spline batch correction (QC-RSC) calibration. Subsequently, metabolites were assessed via principal component analysis (PCA) and partial least squares-discriminant analysis (PLS-DA) to confirm differences and sample reliability [34]. Differentially abundant metabolites (DAMs) were identified based on a fold change (FC) > 2 or <0.5, VIP > 1, and *q* < 0.05 (*t*. test_*p*. value_BHcorrect).

### 2.5. Transcriptome Sequencing and Data Analysis

RNA extraction from young lily leaf samples was carried out using a Plant/Fungi Total RNA Purification Kit (NORGEN: 25800) following the manufacturer’s instructions. The quantity and quality of the isolated RNA were evaluated via a Bioanalyzer 2100 with an RNA 1000 Nano LabChip Kit (Agilent, Santa Clara, CA, USA), ensuring an RNA integrity number (RIN) > 7.0. Transcriptome analysis was performed by LianChuan Biology Technology Company Limited, and paired-end sequencing was performed on an Illumina HiSeq 4000 platform. The sequence integrity was confirmed using FastQC. Trinity 2.4.0 software [35] was used to perform the de novo assembly of the transcriptome. The alignment of all assembled unigenes was carried out against various databases, including nonredundant (Nr) protein, Gene Ontology (GO), SwissProt, KEGG, and eggNOG, using DIAMOND [36], with an E value threshold of <0.00001. Salmon [37] was utilized to assess the expression levels of unique genes based on transcripts per kilobase million (TPM) [38]. The unigenes showing differential expression were pinpointed with a log2 (fold change) > 1 or log2 (fold change) < −1 and an adjusted *p*-value (FDR) < 0.05 via the R tool edgeR [39]. The assessment of gene expression data in short time series involved clustering, comparison, and visualization using STEM V3.11 [40].

### 2.6. Validation of Genes by Quantitative Real-Time PCR

To verify the RNA-seq findings, nineteen genes were chosen for quantitative analysis via qRT-PCR. RNA was isolated from lily leaves of the same group of plants utilized in the RNA-seq experiment. Subsequently, cDNA synthesis was performed by reverse transcribing total RNA using the TRUEscript 1st Strand cDNA Synthesis Kit from Aidlab in Beijing, China. Gene-specific primers (Table S1) were designed via Primer 5 software from Premier Biosoft International in Palo Alto, CA, USA. The qRT-PCR experiments were carried out on an Analytik Jena-q TOWER 2.2 Real-time PCR Detection System from Analytik Jena in Jena, Germany, using SYBR^®^ Green Supermix (Chengdu, China), according to the provided protocol. The qRT-PCRs were replicated three times, both biologically and technically.

### 2.7. Integrated Metabolome and Transcriptome Analyses

Pearson correlation coefficients were computed between the metabolome and transcriptome data. KEGG coenrichment analysis was conducted through the MetWare Cloud (R version 3.5.1, ggplot2 3.3.0), an accessible online platform for data analysis (https://cloud.metware.cn (accessed on 26 October 2023)). A correlation network was constructed using the MetWare Cloud. Correlations with an R^2^ coefficient absolute value higher than 0.8 were chosen.

## 3. Results

### 3.1. Effects of A. gossypii Infection on the Antioxidant Enzyme Activities of Lily

**Figure 1:**
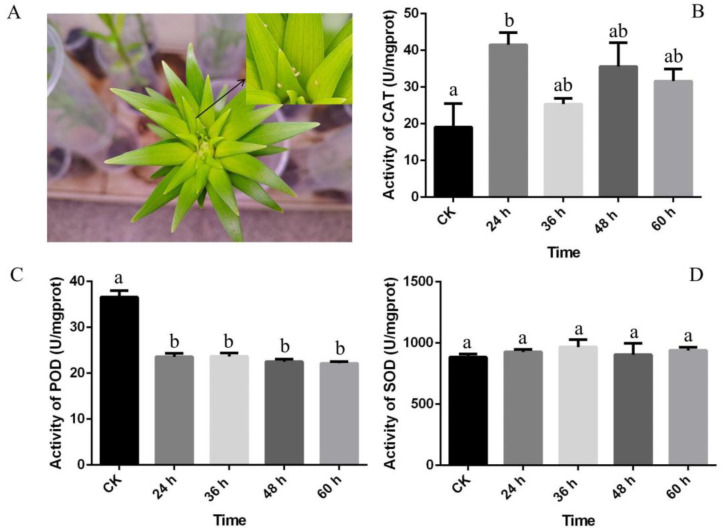
Activities of catalase (CAT), peroxidase (POD), and superoxide dismutase (SOD) at different time-points after aphid attack. (**A**) ‘Fagio’ lily after *A. gossypii* attack. Lily plants were inoculated with *A. gossypii* 30 days after planting. *A. gossypii* infested the plants for 24 h. (**B**–**D**) Activities of CAT, POD, and SOD. The different letters indicate statistically significant differences according to one-way ANOVA, at *p* < 0.05 (a post-hoc Duncan test was used).

Antioxidant enzyme activities were measured to analyze the response of lily leaves to *A. gossypii* infection (Figure 1A). Overall, the results showed a general increase in CAT activity (Figure 1B), whereas POD activity notably decreased after 24 h (Figure 1C). No significant changes were detected in the activity of SOD at the four time points under aphid stress (Figure 1D). In summary, CAT activity was positively correlated with aphid resistance, whereas POD activity was negatively correlated with aphid resistance. There was no significant difference in SOD.

### 3.2. Summary of the Metabolomics of Lily under A. gossypii Infection

To investigate the metabolic alterations resulting from the *A. gossypii* attack, leaf tissues of the lily variety Fangio were inoculated for 0, 24, or 48 h with *A. gossypii* and examined via liquid chromatography-mass spectrometer (LC-MS). In both positive and negative ion modes, 14,572 and 13,634 features were found, respectively. Our metabolomic data revealed 604 metabolites with known structures in lily (Figure S1). These metabolites were further classified into 62 functional categories (Figure S2), with the largest category being carboxylic acids and derivatives (31.79%), followed by fatty acyls (11.59%), flavonoids (5.63%), prenol lipids (4.30%), benzene and substituted derivatives (3.97%), organic oxygen compounds (3.97%), sterol lipids (1.99%), and cinnamic acids and their derivatives (1.82%). A detailed breakdown of all identified metabolites can be found in Table S2.

PCA is often utilized as a multivariate approach for the simultaneous exploration of multiple variables. To gain a comprehensive view of the identified metabolites, PCA was conducted based on the levels of the metabolites. The PCA results revealed that the first two principal components (PCs) accounted for 40.24% of the overall variation (Figure S3). Within the scatter plot, the three sample groups clearly formed separate clusters (specifically, the 24 h, 48 h, and control check (CK) groups were clearly distinguished from each other), indicating unique metabolic characteristics within each respective sample group.

To characterize the primary categories of metabolites related to *A. gossypii* infection, the metabolome samples were divided into three sets (24 h vs. 0 h, 48 h vs. 0 h, and 48 h vs. 24 h). In total, 66, 78, and 65 significant DAMs were identified in the 24 h vs. 0 h, 48 h vs. 0 h, and 48 h vs. 24 h comparisons, respectively (Figure S4). Among the differentially accumulated metabolites, 27 were lipids and lipid-like molecules (such as fatty acyls, prenol lipids, glycerophospholipids, glycerolipids, and sterol lipids), 25 were classified as phenylpropanoids and polyketides (including flavonoids, cinnamic acids and their derivatives, coumarins and their derivatives, and linear 1,3-diarylpropanoids), and 20 were identified as organic acids and derivatives (Table S3).

**Figure 2:**
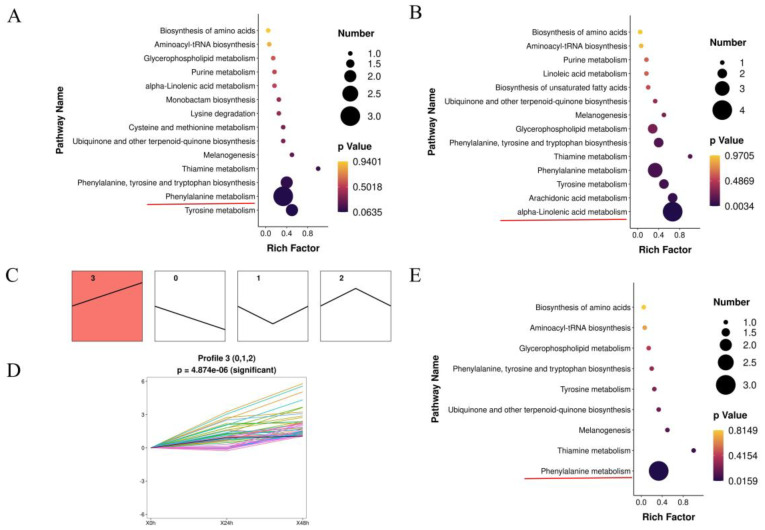
Kyoto Encyclopedia of Genes and Genomes (KEGGs) database enrichment at different time points. (**A**,**B**) KEGG pathway enrichment analysis of differentially abundant metabolites (DAMs) in the lily metabolome induced by *A. gossypii* infestation for 24 h vs. 0 h and 48 h vs. 0 h. (**C**) Time-series metabolomics analysis of significant DAMs induced in lily by *A. gossypii* infestation. The number in the top in the each panel indicates the class. Red panel represents the significantly increased categories, *p* < 0.01. (**D**) The largest significant cluster 3. (**E**) The KEGG pathway for profile 3.

Among the different comparisons, DAMs were strongly associated with phenylalanine metabolism and α-linolenic acid metabolism (Figure 2A,B). To characterize the expression profiles further, the 108 DAMs were hierarchically clustered into four clusters according to changes in stage (Figure 2C). These DAMs were divided into four expression pattern types, namely, the continuous upregulation (cluster 3) (Figure 2D), the continuous downregulation (cluster 0), an initial decrease and then increase (cluster 1), and an initial increase and then decrease (cluster 2). The largest significant cluster, cluster 3 (50 DAMs), was strongly associated with phenylalanine metabolism (Figure 2E).

### 3.3. Transcriptomic Analysis of Lily Responses to A. gossypii Feeding

To explore the global changes in transcriptome expression triggered by *A. gossypii* infestation, a total of nine libraries (comprising three replicates, each for 0, 24, and 48 h) were constructed, yielding approximately 35–52 million valid reads. The GC content of these reads ranged from 50.44% to 53.19% (Table S4). The clean reads were subjected to assembly using Trinity software, resulting in 54,523 unigenes (Table S5) with sizes between 201 and 8451 bp (n50, 1198 bp) (Figure S5) and a mean GC content of 45.44%. Subsequent analyses were conducted using the unique reads.

Leaf transcriptome information was verified by real-time PCR (qRT-PCR) analyses of samples from the same lily batches used for RNA-seq. The expression profiles of nineteen selected differentially expressed genes (DEGs) linked to plant-pathogen interactions, the MAPK signaling pathway, plant hormone signaling, phenylpropanoid, diterpenoid, and isoflavonoid biosynthesis were aligned with the results obtained from RNA-seq (Figure S6). This correspondence suggested the trustworthiness of the RNA-seq data in this study.

**Figure 3:**
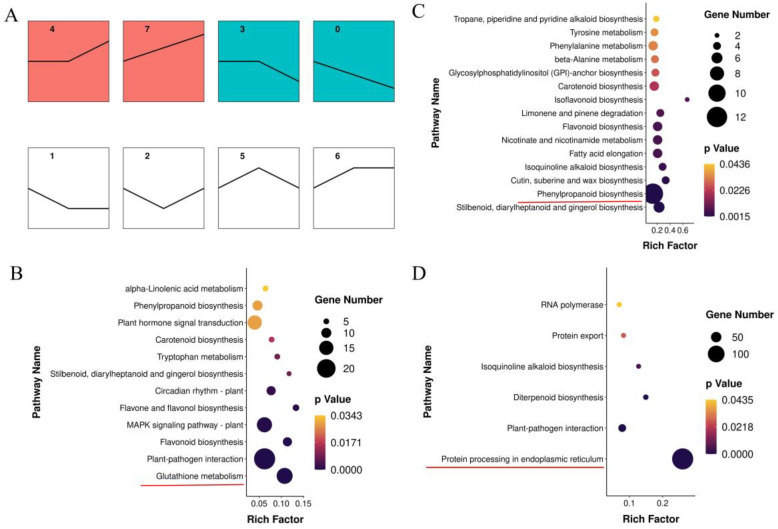
Time-series analysis of differentially expressed genes (DEGs) in lily in response to *A. gossypii* infestation. (**A**) Time-series transcriptomics analysis of significant DEGs induced in lily by *A. gossypii* infestation. The number in the top in the each panel indicates the class. The red panels represent the significantly increased categories, *p* < 0.05. The blue-green panels represent the significantly reduced categories, *p* < 0.05. (**B**–**D**) KEGG pathways for profiles 4, 7, and 3.

In the lily leaves, a total of 3574 DEGs were found following infestation by *A. gossypii* at different time points (24 and 48 h) (Table S6). Similarly, comparisons between 24 or 48 h vs. 0 h and between 48 h vs. 24 h showed 21 DEGs (19 upregulated and 3 downregulated), 3566 DEGs (1115 upregulated and 2451 downregulated), and 2528 DEGs (1325 upregulated and 1203 downregulated) (Tables S7 and S8). KEGG pathway analysis was subsequently performed on all DEGs at different time points to identify the primary metabolic pathways involved (Table S9). The DEGs in the 24 h vs. 0 h and 48 h vs. 0 h comparisons were assigned to 5 and 12 significant KEGG pathways, respectively (*p* < 0.05). The three pathways related to the most genes were involved in protein processing in the endoplasmic reticulum, interactions between plants and pathogens, and diterpenoid biosynthesis. Additionally, the application of the short time-series expression miner (STEM) allowed for the identification of significant temporal expression patterns and the association of genes with these patterns, as well as the comparison of gene behavior across different conditions. To gain insight into the response of the lily transcriptome to *A. gossypii* infestation, an analysis of the total DEGs, using STEM, was conducted. A total of eight key temporal gene expression patterns were identified (Figure 3A, Table S10). Profile 4 (Figure 3B) contained the greatest number of transcripts (348). The upregulated pathways (profiles 4 and 7, Figure 3B,C) were linked to glutathione metabolism, plant-pathogen interactions, the MAPK-signaling pathway–plant, and phenylpropanoid biosynthesis. The downregulated pathways (profiles 0 and 3, Figure 3D) were involved mainly in protein processing in the endoplasmic reticulum.

As a response to stress caused by insects, the activation of downstream pathways by transcription factors (TFs) can offer insight into the molecular mechanisms involved in how plants respond to insect herbivory stress. The significant DEGs included 1318 TFs from 53 families (Table S11, Figure S7), and 58.24% of the TFs belonged to the bHLH, MYB_related, ERF, C3H, NAC, NF-YA, bZIP, NF-YB, GATA, and WRKY families, revealing their key roles in regulating the lily response to *A. gossypii* infestation.

### 3.4. Integrated Analysis of Genes and Metabolites in Lily during A. gossypii Infection

Gene-metabolite interactions were visualized using KEGG pathway maps. One pathway, namely, glutathione metabolism, was significantly enriched in both the transcriptomic and metabolomic data (Figure S8). Furthermore, phenylalanine metabolism and α-linolenic acid metabolism, which were identified via both metabolome (Figure 2A,B) and transcriptome (Figure 3B,C) correlation analyses, could play crucial roles in the biological response to *A. gossypii* infection.

#### 3.4.1. Glutathione Metabolism

**Figure 4:**
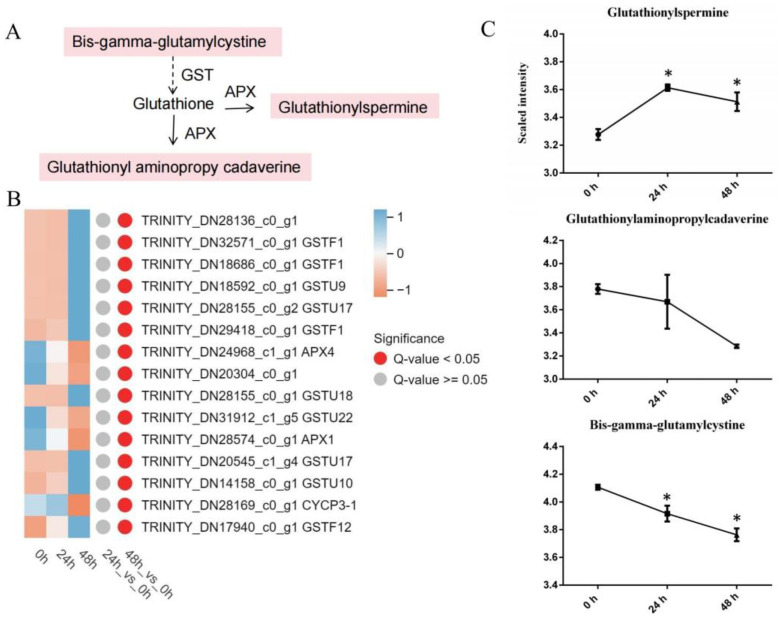
Patterns of gene expression and metabolite levels related to glutathione metabolism induced by *A. gossypii*. (**A**) Pathway schematic. The uppercase letters indicate genes that encode enzymes. The metabolites shaded in pink were measured. The solid arrows represent established biosynthesis steps, while the broken arrows indicate the involvement of multiple enzymatic reactions. (**B**) Heatmap displaying the expression levels of glutathione metabolism genes. A heatmap was generated using the MetWare Cloud (https://cloud.metware.cn (accessed on 19 April 2024)), with the expression quantity normalized to the TPM. (**C**) Abundance of metabolites after *A. gossypii* infestation. The values are log_10_ means ± SDs, *n* = 6. *, *p* < 0.05.

The findings revealed that the DAMs and DEGs enriched in the “glutathione metabolism” pathway displayed uniform expression or abundance trends, with notable variances (*p* value < 0.05) (Figure S8), confirming that the resistance of lily to *A. gossypii* infection was regulated mainly by glutathione metabolism. According to the KEGG pathway analysis of the glutathione metabolism (Table S12), there were 15 DEGs and three DAMs. Among the DEGs (Figure 4B), 12 genes were members of the glutathione S-transferase family, two genes were associated with the ascorbate peroxidase gene family, and one gene belonged to the P-type cyclin gene family. The DAMs (Figure 4C) revealed three compounds, two of which were associated with decreased levels of glutathionyl aminopropyl cadaverine and bis−γ-glutamylcysteine, whereas the level of glutathionylspermine increased. Integrated analysis of the metabolome and transcriptome was utilized to investigate potential genes linked to glutathione metabolism (Figure 4A). Coexpression analysis of DEGs and DAMs revealed a network comprising three DEGs and two DAMs. Among the DEGs, *GSTF1* and *GSTU17* were negatively correlated with glutathionyl aminopropyl cadaverine and bis−γ-glutamylcysteine. *APX4*, glutathionyl aminopropyl cadaverine, and bis−γ-glutamylcysteine were positively correlated.

#### 3.4.2. Phenylalanine Metabolism

**Figure 5:**
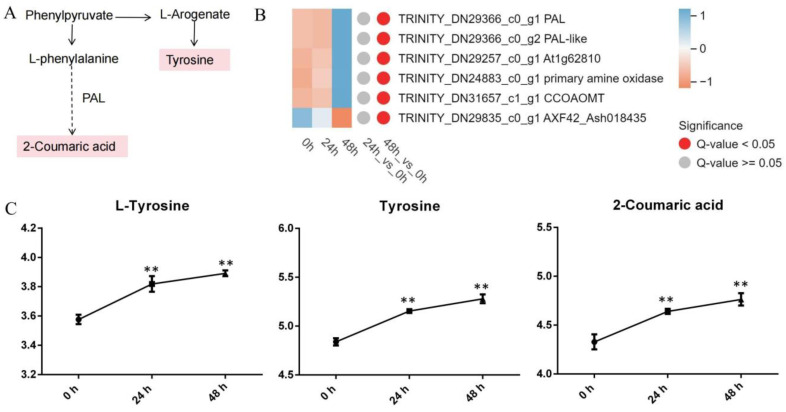
Patterns of gene expression and metabolite levels related to the phenylalanine metabolism induced by *A. gossypii*. (**A**) Pathway schematic. The uppercase letters indicate genes that encode enzymes. The metabolites shaded in pink were measured. The solid arrows represent established biosynthesis steps, while the broken arrows indicate the involvement of multiple enzymatic reactions. (**B**) Heatmap displaying the expression levels of phenylalanine metabolism genes. A heatmap was generated using the MetWare Cloud (https://cloud.metware.cn (accessed on 19 April 2024)), with the expression quantity normalized to the TPM. (**C**) Abundance of metabolites after *A. gossypii* infestation. The values are log_10_ means ± SDs, *n* = 6. **, *p* < 0.01.

The phenylalanine metabolism pathway significantly influences the plant response to biological stress. The analysis of the metabolic and transcriptomic information demonstrated that an increase in gene transcription was linked to an increase in key pathway metabolites (Figure 5). The majority of the DEGs related to phenylalanine metabolism were upregulated (phenylalanine ammonia-lyase (*PAL*), *PAL*-*like* (predicted), *At1g62810*, primary amine oxidase (predicted), and *CCoAOMT*), except for *AXF42_Ash018435* (hypothetical protein) (Figure 5B). Across the two comparison groups (24 h vs. 0 h and 48 h vs. 0 h) under *A. gossypii* feeding, three differentially abundant metabolites (L-tyrosine, tyrosine, and 2-coumaric acid) were found to be significantly enriched in the phenylalanine metabolism pathway (Figure 5C). In lily plants, *A. gossypii* feeding activated *PAL* and *CCoAOMT* expression and increased L-tyrosine, tyrosine, and 2-coumaric acid synthesis in the resistant genotype.

The lignin pathway and flavonoid pathway are major branches of phenylalanine metabolism. Flavonoids are important secondary metabolites that usually play a decisive role in preventing insect infestation. The pathways involved in the synthesis of flavonoids included 17 metabolites (seven quercetins, two delphinidins, two isorhamnetins, rutin, and benzopyran) (Table S3). *CCoAOMT* genes (Figure 5B) are exclusive to the lignin pathway. These results suggest that the lignin pathway also plays a role in regulating aphid resistance in lilies.

#### 3.4.3. α-Linolenic Acid Metabolism

**Figure 6:**
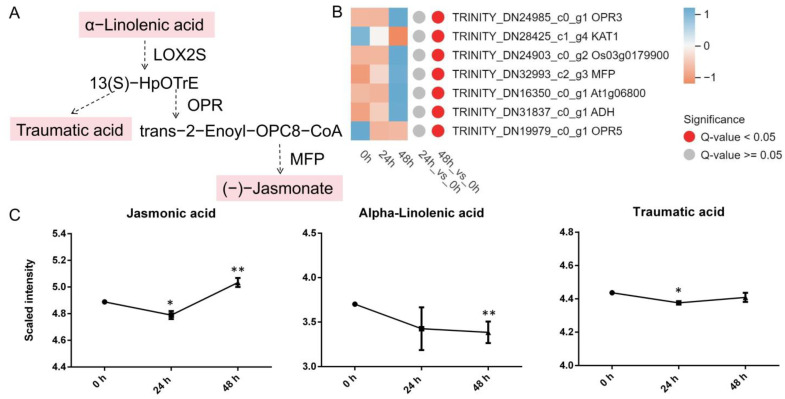
Patterns of gene expression and metabolite levels related to α-linolenic acid metabolism induced by *A. gossypii*. (**A**) Pathway schematic. The uppercase letters indicate genes that encode enzymes. The metabolites shaded in pink were measured. Broken arrows indicate the involvement of multiple enzymatic reactions. (**B**) Heatmap displaying the expression levels of α-linolenic acid metabolism genes. A heatmap was generated using the MetWare Cloud (https://cloud.metware.cn (accessed on 22 April 2024)), with the expression quantity normalized to the TPM. (**C**) Abundance of metabolites after *A. gossypii* infestation. The values are log_10_ means ± SDs, *n* = 6. *, *p* < 0.05. **, *p* < 0.01.

Most DEGs involved in α-linolenic acid metabolism were upregulated (*OPR3*, *Os03g0179900*, *MFP*, *At1g06800*, and *ADH*), except for the genes *KAT1* and *OPR5* (Figure 6B). Across the two comparison groups (24 h vs. 0 h and 48 h vs. 0 h) under *A. gossypii* feeding, three differentially abundant metabolites (jasmonic acid (JA), α-linolenic acid, traumatic acid) were found to be significantly enriched in the α-linolenic acid metabolism pathway. Among these metabolites, α-linolenic acid significantly decreased at 48 h. Traumatic acid significantly decreased at 24 h, while JA significantly increased under aphid stress (Figure 6C). JA was positively correlated with *OPR3* and *Os03g0179900*.

## 4. Discussion

Our results demonstrated that the connection between *A. gossypii* infestation and the defensive response of lily plants manifested in antioxidant reactions, phenylpropane and flavonoid biosynthesis, and α-linolenic acid metabolism.

### 4.1. Antioxidant Reactions in Lily after Cotton Aphid Infection

Most of the defense enzymes involved in the plant anti-insect defense response showed a positive correlation, while some showed a negative correlation or no obvious correlation. When cotton (*Gossypium hirsutum*) was fed *A. gossypii*, *Spodoptera litura Fabricius*, or *Lygus lucorum*, the CAT, POD, and SOD activities of the cotton leaves increased, which was positively correlated with insect resistance [41,42]. This finding is in accordance with our results on the increase in CAT activity in lily leaves under *A. gossypii* stress. In contrast, our study revealed that POD activity decreased and that SOD activity did not change significantly in lily leaves under *A. gossypii* stress. However, under *L. lucorum* feeding, there was no significant difference in CAT activity between the leaves of jujube, peach, cherry, and grape plants [43]. Under *L. lucorum* feeding, the SOD activity in cherry leaves decreases, which is negatively correlated with insect resistance [43].

Glutathione serves as a necessary cofactor for multiple antioxidant enzymes, including glutathione peroxidases and glutathione S-transferases (GSTs) [44]. The analysis showed that *A. gossypii* infestation increased the *GST* gene expression in lily leaves (Figure 4B). In accordance with findings by Sytykiewicz [45], the upregulation of the *gst9*, *gst11*, *gst16*, *gst31,* and *gst38* genes was detected in maize seedlings under stress induced by *Sitobion avenae* and *Rhopalosiphum padi*. Moran et al. also noted a comparable trend in *Arabidopsis thaliana* L. after infestation with *Myzus persicae*, where the *gst1* and *gst11* genes presented nearly threefold and fivefold increases in expression, respectively [46].

### 4.2. Phenylalanine Metabolism and Flavonoid Biosynthesis Pathways Involved in the Response to Aphid Stress

Plants regulate the synthesis of different metabolites by regulating gene expression and changes in enzyme activity in the main branches of the phenylpropanoid metabolism pathway, namely, the lignin pathway and flavonoid pathway, to respond to disease and insect feeding. Our transcriptomic analysis revealed that most genes related to phenylalanine metabolism were notably upregulated during the late phase (48 h) of *A. gossypii* infection. Numerous studies have established the significant impact of PAL activity on plant resistance, with protein activity emerging as a key physiological index for determining plant resistance [47]. Plants often exhibit increased *PAL* expression in response to pests and pathogens. For instance, in *Brachypodium*, sorghum (*Sorghum bicolor*) hybrids, and rice (*Oryza sativa* L. cv. *Gigante Vercelli*), *PAL* expression is elevated following fungal infections [48,49,50]. The resistance of sorghum to sugarcane aphids is attributed to the *SbPAL* gene [51]. In our study, *PAL*, which is involved in phenylalanine metabolism, was upregulated, which is consistent with previously reported findings (Figure 5A,B). *A. gossypii* feeding on lily activated *PAL* expression in the resistant genotype.

Flavonoid biosynthesis, a process that occurs downstream of phenylpropane metabolism, has garnered extensive attention for its protective role in plant resistance to different biotic and abiotic stresses [52]. Specialist aphids are thought to have evolved mechanisms to tolerate the toxic effects of secondary metabolites [53]. Flavonoids, on the other hand, appear to be interesting candidate compounds conferring resistance to aphids. The presence of flavonoids in apple plants has been shown to have insecticidal effects on the woolly apple aphid *Eriosoma lanigerum*, which causes mortality in both nymphs and wingless adults [54]. Flavonoids are vital for aphid mortality and provide resistance to corn leaf aphids on sorghum plants [55]. Chlorogenic acid, quercetin, and ferulic acid can increase significantly after groundnuts are invaded by *Helicoverpa armigera* and *Aphis craccivora*, which can improve the insect resistance of groundnuts [56]. Quercetin and rutin can influence aphids (*Acyrthosiphon pisum*, *M. persicae,* and *R. padi*) to suck plant juice through their mouthparts [57]. The addition of naringenin and quercetin to the artificial feed of pea aphids (*A. pisum*) significantly reduced their fertility and significantly increased their developmental duration and adult mortality [58]. The detrimental impact of flavonoids on insects has also been validated in other studies [59]. Similarly, Simmonds [60] noted that insect feeding and oviposition behavior are influenced by flavonoids. In our study, among the 108 DAMs, 17 flavonoids were differentially accumulated in the resistant lily cultivar Fangio in response to infestation by *A. gossypii* (Table S3). Similarly, feeding by the grain aphid *S. avenae* increased the amount of flavonoids and flavones in resistant winter triticale plants [61]. The combination of these findings with our own results underscores the effectiveness of flavonoid metabolites in providing defense against aphids, suggesting their potential use as bioinsecticides in integrated pest management programs.

### 4.3. α-Linolenic Acid Metabolism Participates in the Stress Response

Research on plant stress resistance has focused on α-linolenic acid metabolism, which is the starting point of JA synthesis. When plants are damaged by pests or diseases, JA levels increase rapidly [62]. This result is consistent with our finding, that aphids significantly increased the JA content in lily leaves (Figure 6C). Exogenous MeJA can improve the activity of protease inhibitors in plants to achieve insect resistance. Subsequent relevant studies have shown that JAs are nontoxic to insects but can induce plants to synthesize a variety of insect-resistant compounds, including insect repellent volatiles, toxic secondary metabolites, and protease inhibitors (jasmonates) [62]. JA can induce the activation of defense genes and the synthesis of secondary compounds, as well as improve the stress resistance of plants [63].

*OsOPR7.1* was proven to improve resistance to *Xanthomonas oryzae* pv. *oryzicola* by increasing JA synthesis in rice [64]. However, *TaOPRIII-7* gene silencing increased the resistance of wheat to greenbugs. Studies have shown that the activity of the *OPR* gene is regulated by plant hormones and various stresses, and the resistance of plants is improved through JA and other pathways [65]. In Arabidopsis, *OPR3* is involved in the biosynthetic pathway of JA, regulating its resistance to *Bradysia impatiens* and *Alternaria brassicicola* [66]. In rice, the overexpression of *OPR3* increased the JA content and increased resistance to *Chilo suppressalis*, but the plants were susceptible to *Nilaparvata lugens* [67]. In this study, *OPR3* was upregulated under aphid stress and was positively correlated with JA. According to the above studies on wheat, rice, and Arabidopsis, the anti-aphid effect of *OPR* may be regulated by a complex signaling network.

In the future, we can further study the resistance of lily aphids to antioxidant reactions, lignin and flavonoid biosynthesis, and hormone signaling pathways. For instance, we will focus on measuring the contents of JA and SA and analyzing the expression levels of genes related to SA and JA synthesis/signaling pathways to further elucidate the antagonistic interactions between SA and JA that fine-tune lily defense responses against aphid stress. Studying the variances we noted in lily in comparison with findings from other plants could enhance our comprehension and offer perspectives on leveraging pathway networks to achieve resistance to lily aphids and other thorn-sucking pests in other plants.

## 5. Conclusions

To summarize, the combined analysis of antioxidant enzyme, transcriptome, and metabolome produced extensive data on the protection mechanisms of lily plants against *A. gossypii* invasion. The defensive reactions involve glutathione metabolism, phenylalanine metabolism, and α-linolenic acid metabolism. The genetic components and metabolic frameworks identified from this investigation offer fresh perspectives on lily defenses, and these discoveries may aid in the development of lily varieties that are less susceptible to aphids.

## Data Availability

The transcriptomics dataset is available as Bioproject: PRJNA1104790. Run Accessions: SRR29090846-SRR29090854. The original contributions presented in this study are included in the Supplementary Material, and further inquiries can be directed to the corresponding author.

## Supplementary Materials

The following supporting information can be downloaded at: https://www.mdpi.com/article/10.3390/genes15091124/s1, Figure S1: Hierarchical clustering of metabolite expression profiles in *A. gossypii*-infected and control lily leaves; Figure S2: Classes of identified metabolites. The 604 metabolites were classified into 11 different chemical classes (pink circles), and the other colored circles indicate 62 functional categories; Figure S3: Principal component analysis (PCA) of all metabolites detected in leaf. PCA between different treatment groups. AD_24h represents *A. gossypii*-infected for 24 h, AD_48h represents *A. gossypii*-infected for 48 h, CK represents samples without *A. gossypii* infection, QC is a quality control sample; Figure S4: Venn diagram showing the differentially abundant metabolites (DAMs) among different time points vs. 0 h; Figure S5: The assembled transcript length distribution; Figure S6: The mRNA expression levels of 19 selected genes were compared using RNA-seq (solid squares) and qPCR (solid circles). The qRT-PCR data were normalized to those of the housekeeping gene *GAPDH*. The values are the means ± SEs; *n* = 3. All the expressions are normalized to 0 h. More detailed information about these genes can be found in Table S1; Figure S7: Distribution of transcription factor (TF) families among the differentially expressed genes (DEGs); Figure S8: Combined Kyoto Encyclopedia of Genes and Genomes database analysis of DAMs and DEGs; Table S1: Primer pairs utilized for quantitative real-time PCR of genes; Table S2: Details of all detected metabolites; Table S3: The components of the lipids and lipid-like molecules, phenylpropanoids and polyketides, and organic acids and derivatives; Table S4: Summary of the transcriptome dataset; Table S5: Genes detected in all samples. The levels of gene expression are indicated using TPM. All samples were harvested at 0, 24, and 48 h following *A. gossypii* infestation; -: no annotation; Table S6: DEGs in lily leaves in response to *A. gossypii* feeding at different time points; Table S7: All upregulated DEGs in lily leaves induced by *A. gossypii* for 24 or 48 h with a twofold cutoff change relative to the control; Table S8: All downregulated DEGs in lily leaves induced by *A. gossypii* for 24 or 48 h with a twofold cutoff change relative to the control; Table S9: KEGG pathway enrichment analysis of DEGs in the lily transcriptome following exposure to *A. gossypii* for different periods; Table S10: Overrepresentation analysis of significant profile using short time-series expression miner (STEM) analysis tool to identify metabolic pathways that are being regulated; Table S11: Differentially expressed transcription factors in lily leaves in response to *A. gossypii* feeding; Table S12: The differentially expressed genes and expressed metabolites in the KEGG pathway of glutathione metabolism.
