# Supplementary material for: Antioxidant Enzyme, Transcriptomic, and Metabolomic Changes in Lily (Lilium spp.) Leaves Induced by Aphis gossypii Glover
Source: Genes (Basel). 2024 Aug 26;15(9):1124. doi: 10.3390/genes15091124 (PMC11431739; doi:10.3390/genes15091124)
Supplement: Supplementary file 1 [file genes-15-01124-s001.zip › Supplementary figures S1-S8.pdf]

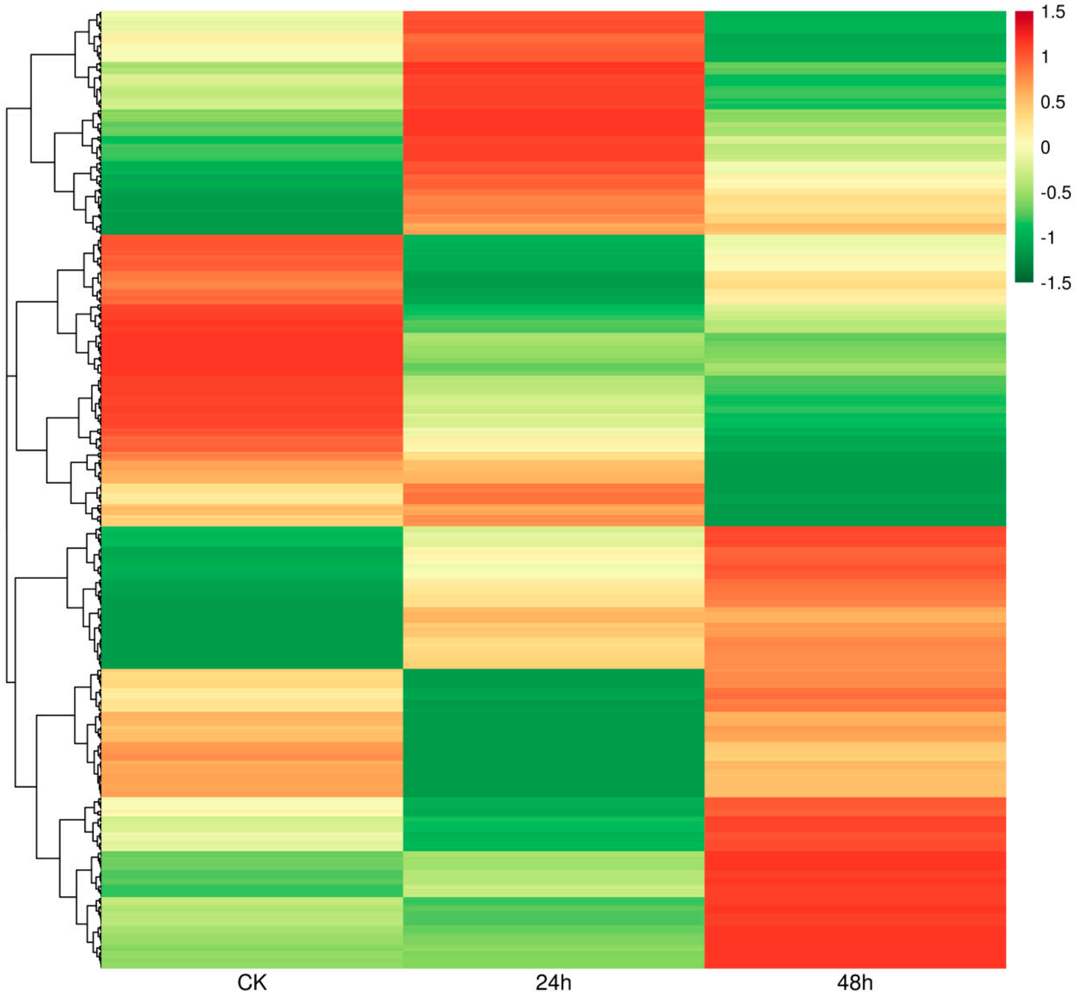

Supplementary Figure S1. Hierarchical clustering of metabolite expression profiles in *A. gossypii*−infected and control lily leaves.

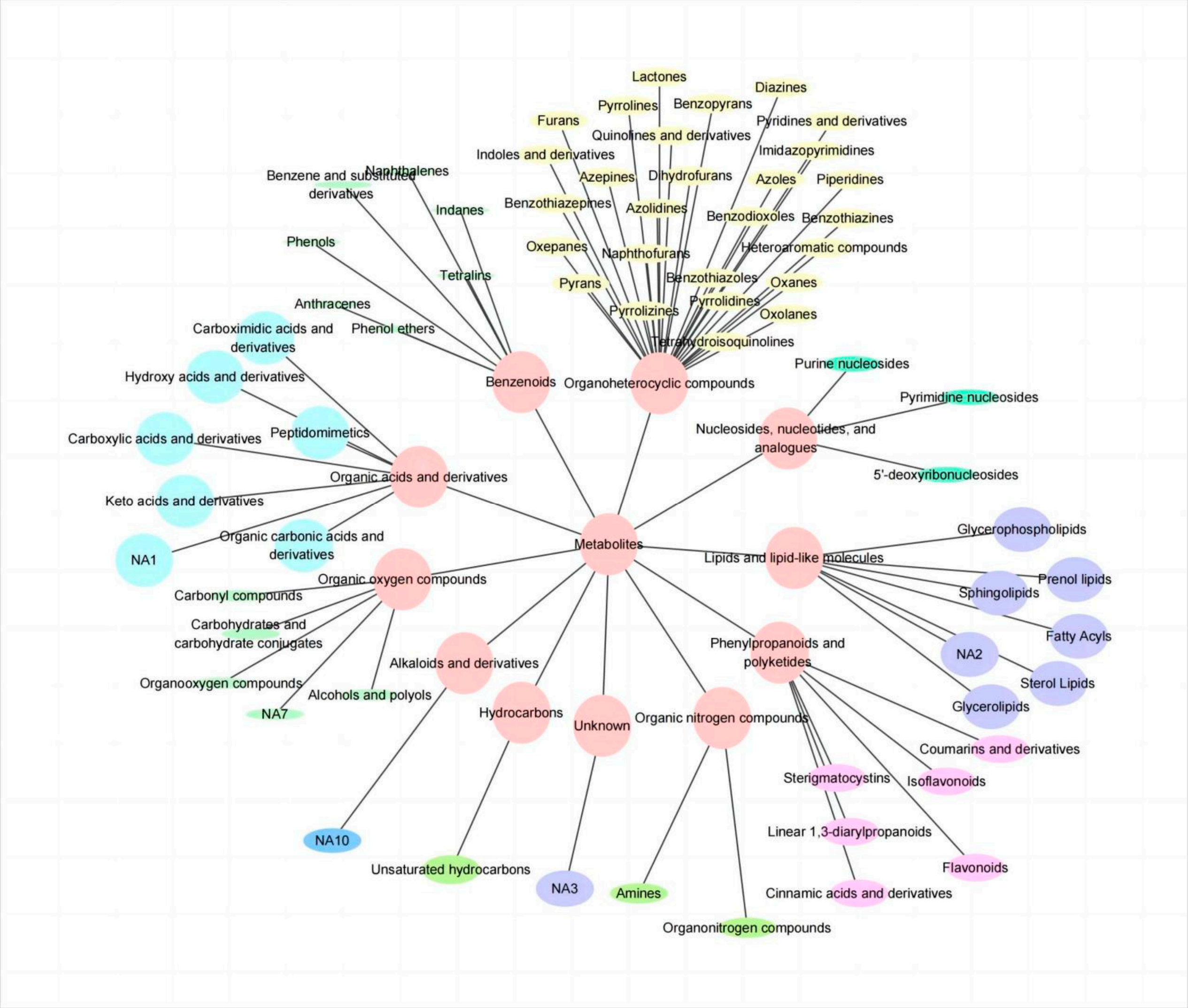

Supplementary Figure S2. Classes of identified metabolites. The 604 metabolites were classified into 11 different chemical classes (pink circles), and the other colored circles indicate 62 functional categories.

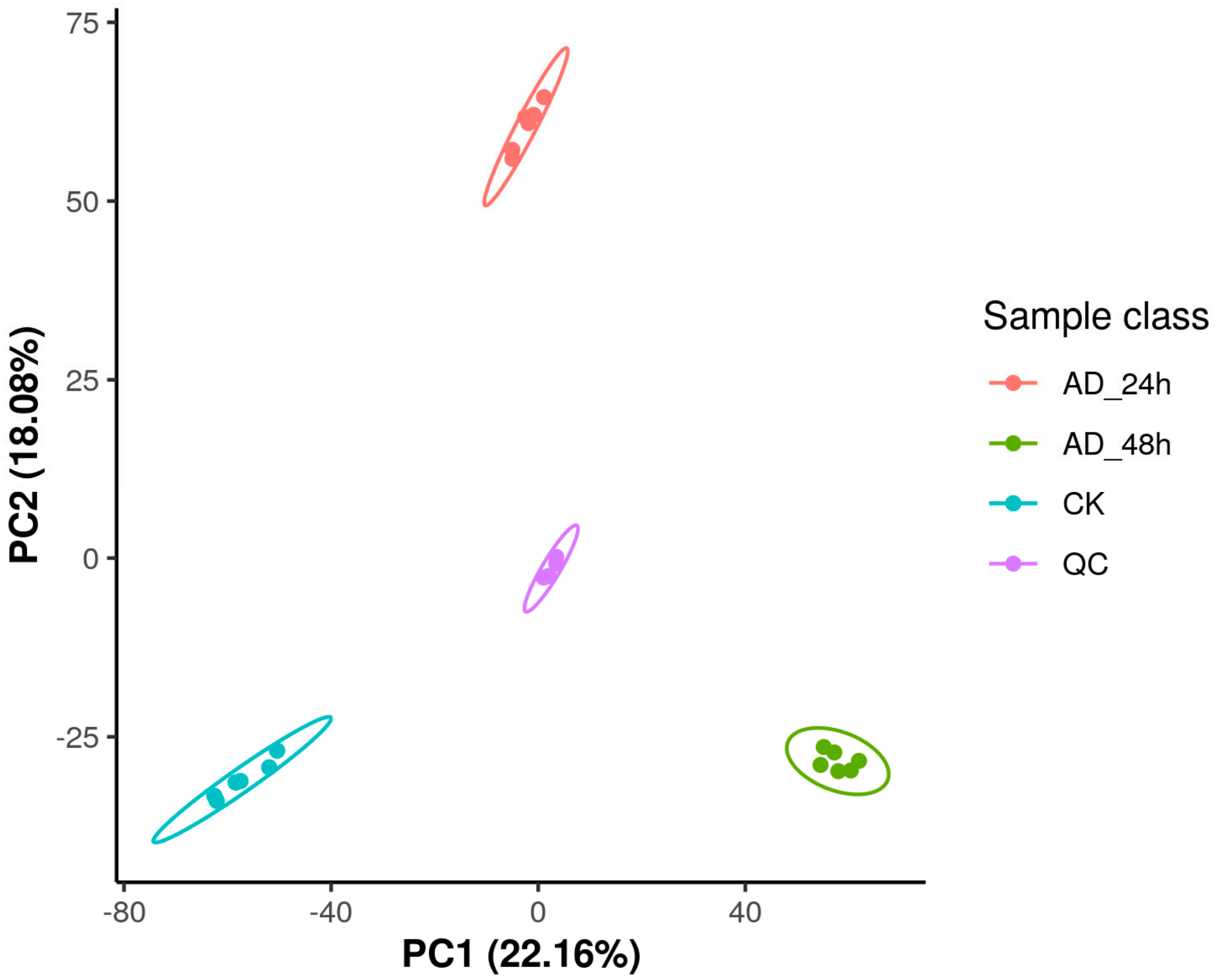

Supplementary Figure S3. Principal component analysis (PCA) of all metabolites detected in leaf. PCA between different treatment groups, AD_24h represents *A. gossypii*-infected for 24 h, AD_48h represents *A. gossypii*−infected for 48 h, CK represents without *A. gossypii*−infected, QC is a quality control sample.

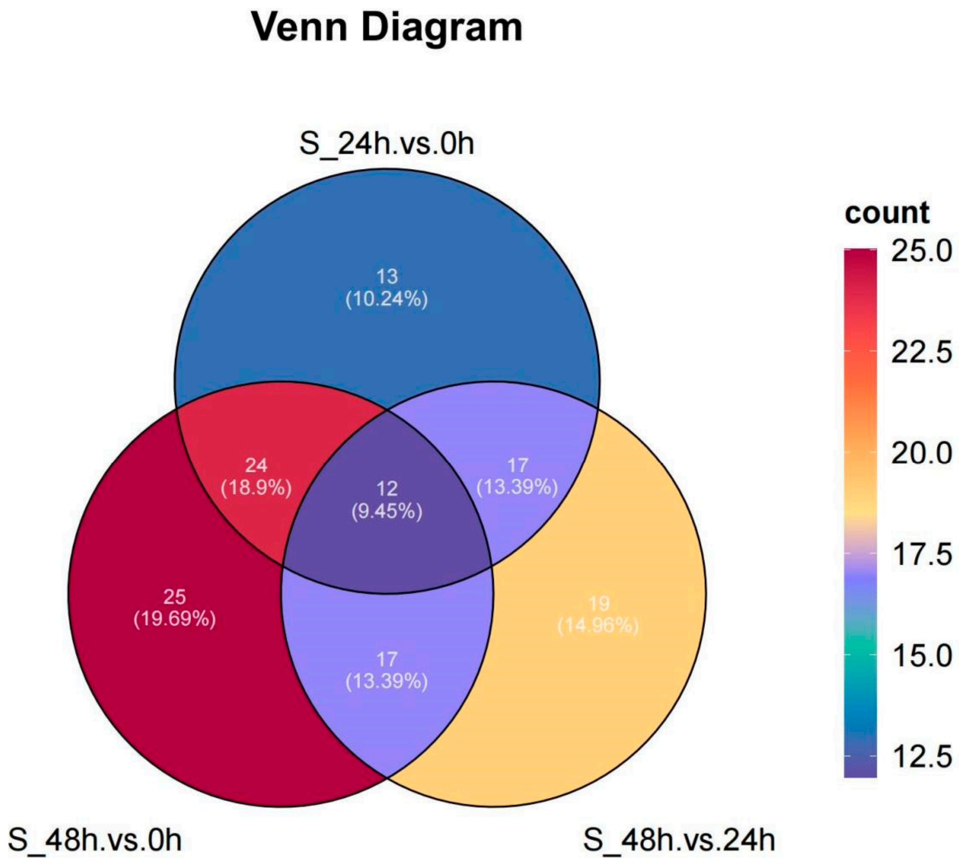

Supplementary Figure S4. Venn diagram showing the differentially abundant metabolites (DAMs) among different time points vs. 0 h

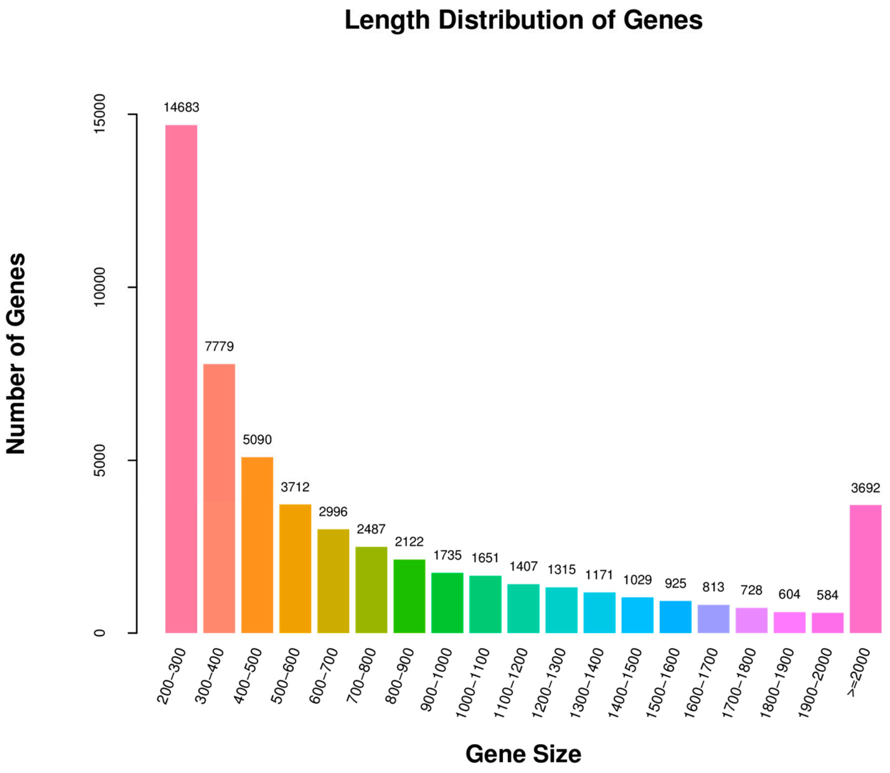

Supplementary Figure S5. The assembled transcript length distribution.

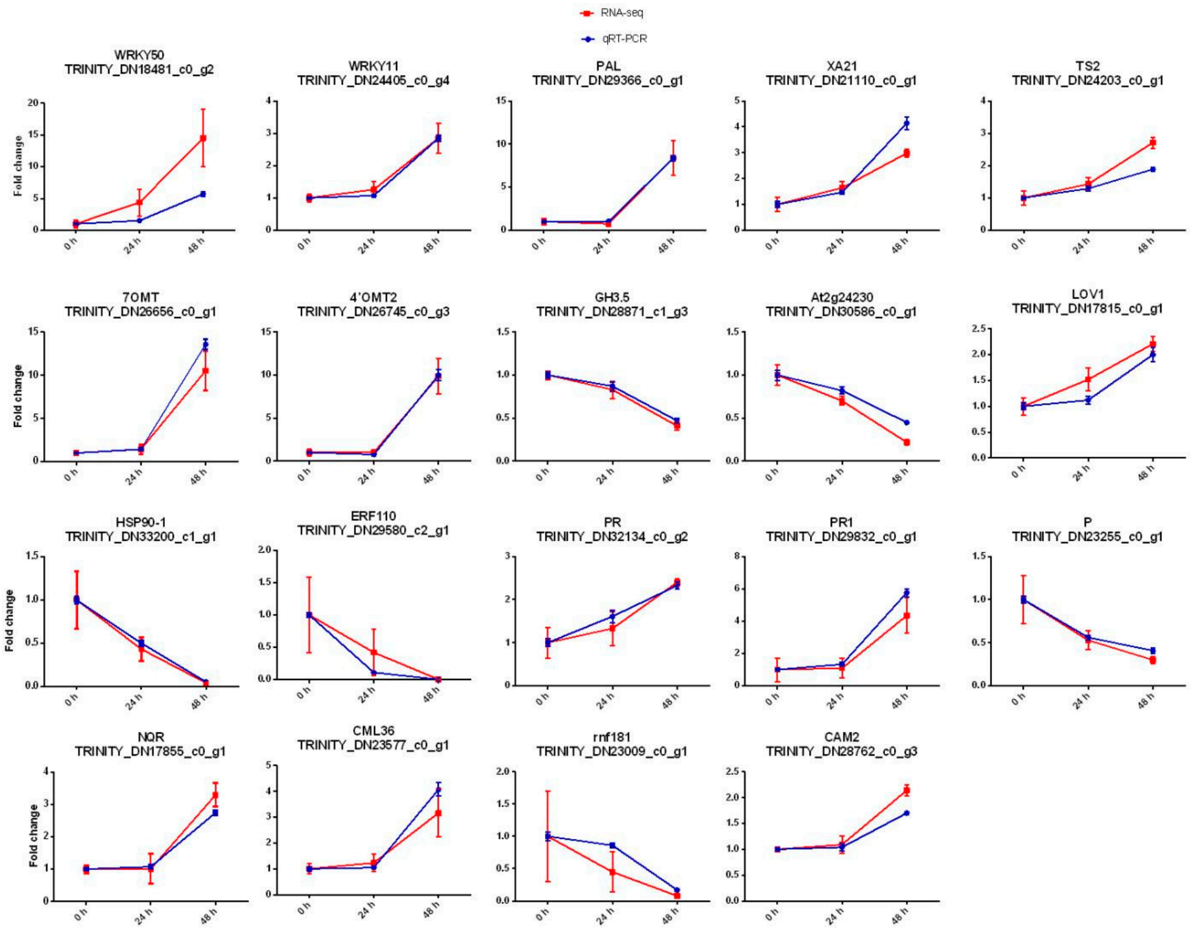

Supplementary Figure S6. The mRNA expression levels of 19 selected genes were compared using RNA−seq (solid squares) and qPCR (solid circles). The qRT−PCR data were normalized to those of the housekeeping gene *GAPDH*. The values are the means ± SEs; *n* = 3. All the expressions are normalized to 0 h. More detailed information about these genes can be found in **Table S1**.

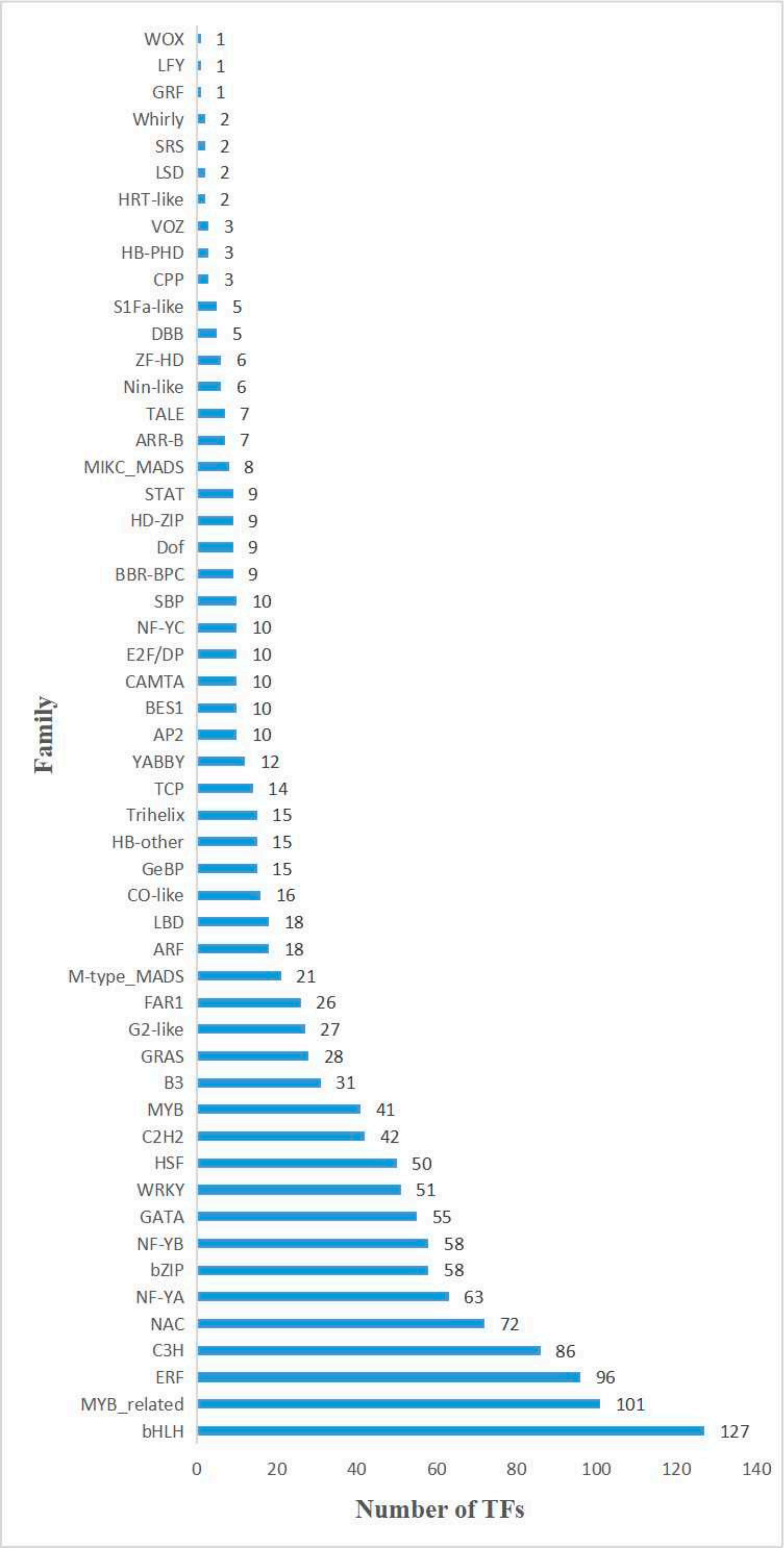

Supplementary Figure S7. Distribution of transcription factor (TF) families among the differentially expressed genes (DEGs).

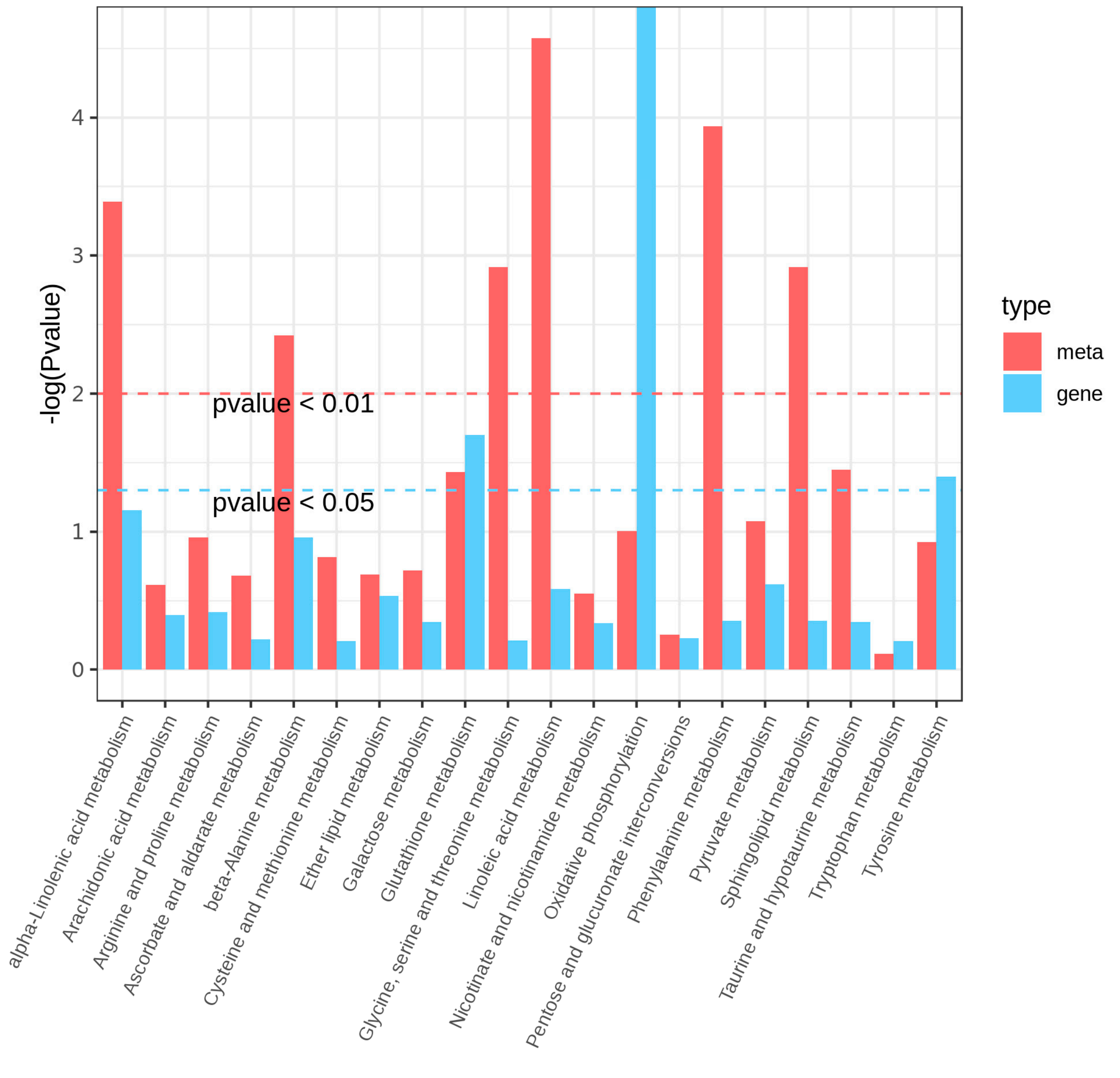

Supplementary Figure S8. Combined Kyoto Encyclopedia of Genes and Genomes database analysis of DAMs and DEGs.
